# Lower limb kinematic, kinetic, and EMG data from young healthy humans during walking at controlled speeds

**DOI:** 10.1038/s41597-021-00881-3

**Published:** 2021-04-12

**Authors:** Luís Moreira, Joana Figueiredo, Pedro Fonseca, João P. Vilas-Boas, Cristina P. Santos

**Affiliations:** 1 Center for MicroElectroMechanical Systems (CMEMS), University of Minho, 4800-058 Guimarães, Portugal; 2 Porto Biomechanics Laboratory (LABIOMEP), University of Porto, 4200-450 Porto, Portugal; 3 Faculty of Sport, CIFI2D, and Porto Biomechanics Laboratory (LABIOMEP), University of Porto, 4200-450 Porto, Portugal

**Keywords:** Biological physics, Biomedical engineering

## Abstract

Understanding the lower limb kinematic, kinetic, and electromyography (EMG) data interrelation in controlled speeds is challenging for fully assessing human locomotion conditions. This paper provides a complete dataset with the above-mentioned raw and processed data simultaneously recorded for sixteen healthy participants walking on a 10 meter-flat surface at seven controlled speeds (1.0, 1.5, 2.0, 2.5, 3.0, 3.5, and 4.0 km/h). The raw data include 3D joint trajectories of 24 retro-reflective markers, ground reaction forces (GRF), force plate moments, center of pressures, and EMG signals from *Tibialis Anterior*, *Gastrocnemius Lateralis*, *Biceps Femoris*, and *Vastus Lateralis*. The processed data present gait cycle-normalized data including filtered EMG signals and their envelope, 3D GRF, joint angles, and torques. This study details the experimental setup and presents a brief validation of the data quality. The presented dataset may contribute to (*i*) validate and enhance human biomechanical gait models, and (*ii*) serve as a reference trajectory for personalized control of robotic assistive devices, aiming an adequate assistance level adjusted to the gait speed and user’s anthropometry.

## Abstract (summary)

**Table:** 

Measurement(s)	walking • Lower Extremity
Technology Type(s)	motion capture • electromyography • force platform
Factor Type(s)	sex • age • height • mass • leg length • foot length
Sample Characteristic - Organism	Homo sapiens

Machine-accessible metadata file describing the reported data: 10.6084/m9.figshare.13169348

## Background & Summary

Walking appears to be the most performed human motor task^1^. However, healthy human gait may be compromised by neurological diseases (such as stroke and spinal cord injury)^2,3^. Robotic assistive devices (namely, active orthosis and exoskeletons) have been recommended to foster repetitive gait rehabilitation sessions for patients with locomotor impairments that lost the ability to walk. Typically, robotic devices endow control architectures that require reference trajectories (e.g., joint angles, torques, or muscle activations) to determine the amount of needed assistance or to define a target walking pattern^4^. These reference trajectories are commonly obtained from public walking datasets from healthy users’ self-selected speeds.

Moreover, control architectures may include objective assessment tools of the patient’s disability level as a fundamental point for developing personalized rehabilitation strategies oriented to the patient’s locomotion condition^5^. Kinematic, kinetic, and electromyography (EMG) data are commonly used to describe the human locomotion condition^6^. Typically, clinical studies distinguish pathological and healthy gait by using these walking data collected at healthy users’ self-selected speeds, available in the literature^7–9^.

Nonetheless, participants with lower limb impairments present slower speeds when compared to the healthy gait at self-selected speeds^10^. Therefore, the validity of applying reference trajectories in robotic devices and the pathological *vs* healthy gait pattern distinction may be compromised due to the potential bias that can be introduced by the speed differences in the gait dynamics^4,11–14^. Additionally, the comprehension of the body mass and height effects on lower limb biomechanical parameters is fundamental to determine changes in the gait pattern^4,13,15–17^. Thus, the provision of multimodal walking datasets oriented to the anthropometry and walking speed conditions of each participant has been highlighted.

Although the high number of performed gait studies, there are still a few available multimodal walking datasets. Furthermore, most of them present the following shortcomings. First, only provide the average and the standard deviation of the data for a single gait cycle for all the participants that participated in each study, not providing the raw data^12^. In this manner, it is not possible to access the walking data considering the anthropometry and speed of each participant. Additionally, some available datasets only foster data for healthy self-selected speeds^5,12,18–20^. On the other hand, some datasets do not provide EMG data, not being suitable for applications that depend on EMG information^19–21^. Despite being suitable for several applications, the usefulness of these datasets can be restricted.

In this study, we present a multimodal walking dataset containing EMG data from four muscles (*Tibialis Anterior* (TA), *Gastrocnemius Lateralis* (GAL), *Biceps Femoris* (BF), and *Vastus Lateralis* (VL)), 3D ground reaction forces (GRFs), Center of Pressures (CoPs), and joint angles and torques of the lower limb joints (hip, knee, and ankle) along with the pelvis segment. The data were collected from sixteen healthy participants performing walking trials at seven controlled speeds (1.0, 1.5, 2.0, 2.5, 3.0, 3.5, and 4.0 km/h, corresponding to 0.28, 0.42, 0.56, 0.69, 0.83, 0.97, and 1.1 m/s, respectively) on a flat surface. The gait speed selection was based on the (***i***) preferred speed of the most incident population with gait impairments and (***ii***) comfortable speed of healthy participants^10,22^. Since the joint biomechanics during the walking motion depend on gender, body height, and mass, the proposed dataset tackles an equal gender distribution with a wide range of body heights (ranging from 1.51 to 1.83 m) and mass (ranging from 52.0 to 83.7 kg)^23,24^. Based on the best knowledge of the authors, this is the first study that presents a database that gathers anthropometric information, synchronized EMG, 3D GRF, CoPs, and angles and torques data of the lower limb joints along with the pelvis segment under controlled speeds.

The proposed multimodal walking dataset may contribute to (***i***) the development of biomechanical gait models scaled to variable anthropometric parameters and speeds; (***ii***) assess the human locomotion conditions based on lower limb kinematic, kinetic, and EMG interrelation at controlled speeds; (***iii***) perform a biomechanical analysis of gait and muscle activation patterns during speed variation; and (***iv***) serve as reference trajectories for robotic devices, aiming an adequate assistance level adjusted to the speed and user’s anthropometry.

## Methods

### Participants

Healthy participants were approached through a mailing list across the University of Minho community describing the study goal, protocol, and duration. The participants were formally recruited and selected according to a set of inclusion criteria, as follows: (***i***) healthy locomotion; (***ii***) more than 18 years old; (***iii***) body mass ranging from 45 to 90 kg; and, (***iv***) body height ranging from 1.50 to 1.90 m. The participants disclaimed a history of injuries or musculoskeletal dysfunctions. These criteria were followed to produce a dataset with a stratified anthropometric distribution while considering possible gender biomechanical differences^23–25^. In this connection, sixteen healthy participants (8 males and 8 females) were involved in the study. They present a mean age of 23.8 ± 2.02 years, a mean body mass of 67.5 ± 10.8 kg, and a mean body height of 1.69 ± 0.108 m, as presented in Table 1. Before undergoing the experiments, all participants provided written and informed consent, according to the ethical conduct defined by the University of Minho Ethics Committee that follows the standard set by the declaration of Helsinki and the Oviedo Convention (CEICVS 006/2020). Moreover, participants also provided written consent to publish identifiable images.

**Table 1:** Anthropometric information of the participants.

ID	Gender	Body Height (m)	Body Mass (kg)	Age (years)	Leg Length (m)	Foot Length (m)
1	Male	1.81	66.2	25	0.84	0.28
2	Female	1.56	54.4	23	0.69	0.24
3	Female	1.57	53.5	26	0.70	0.24
4	Male	1.71	81.7	23	0.79	0.27
5	Male	1.79	74.4	24	0.82	0.27
6	Female	1.65	60.0	21	0.74	0.24
7	Female	1.54	58.4	21	0.66	0.21
8	Male	1.72	75.8	23	0.77	0.27
9	Male	1.80	83.7	26	0.81	0.27
10	Male	1.82	76.8	25	0.81	0.26
11	Female	1.51	52.0	23	0.67	0.25
12	Male	1.83	73.4	25	0.82	0.23
13	Female	1.61	53.5	27	0.70	0.21
14	Female	1.60	65.3	26	0.71	0.26
15	Female	1.70	69.3	20	0.75	0.26
16	Male	1.79	81.9	22	0.79	0.27

### Instrumentation & data collection

**Fig. 1:**
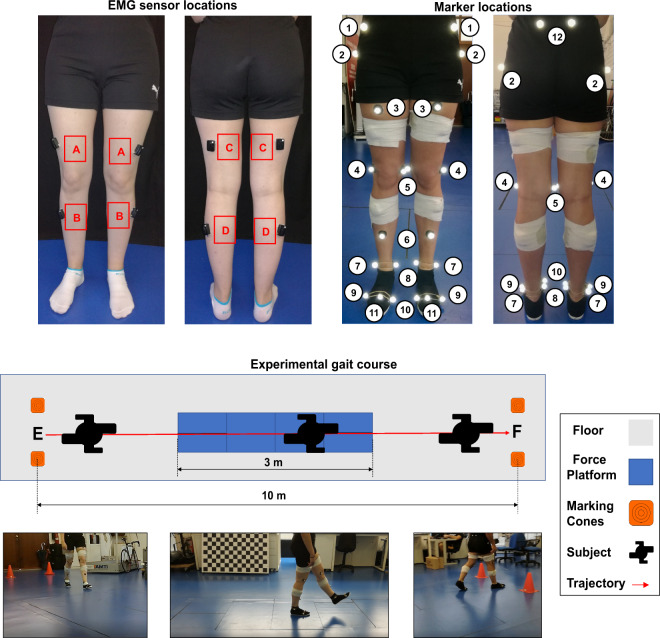
EMG sensors attached to the participant’s skin: (**A**) - VL, (**B**) - TA, (**C**) - BF, and (**D**) - GAL. Retro-reflective markers: 1 - Anterior Superior Iliac Spine (ASIS), 2 - Trochanter (TROC), 3 - Thigh (TH), 4 - Lateral Knee (LK), 5 - Medial Knee (MK), 6 - Shank (SK), 7 - Lateral Ankle (LA), 8 - Medial Ankle (MA), 9 - Foot Metatarsal 5 (FM5), 10 - Foot Metatarsal 1 (FM1), 11 - Foot Metatarsal 2 (FM2), 12 - Posterior Superior Iliac Spine (PSOS). Data acquisition scenario, where E and F represent the beginning and the end position of the recording, respectively.

Data were collected at Porto Biomechanics Laboratory (LABIOMEP). The participants were instrumented with a surface EMG system and lower body motion-capture system, as illustrated in Fig. 1. The participants were asked to wear tight shorts and a standard type of tennis shoes that properly fits the user’s feet.

A surface 8-channel wireless EMG system (Trigno – Delsys, Massachusetts, United States of America), acquiring at 2000 Hz, provided the EMG signals from four lower limb muscles, namely, TA, GAL, BF, and VL of both lower limbs (as presented in Fig. 1). Prior to the EMG sensors’ placement, the skin above each muscle was gently cleaned with alcohol wipes to remove skin and dirt oils. Once cleaned, the eight EMG electrodes (Trigno – Delsys, Massachusetts, bipolar configuration with an interelectrode distance of 10 mm) were attached to the skin with double-sided adhesive tape, following the SENIAM recommendations^26^ (Fig. 1). The quality of all EMG signals was checked, and the location of the sensors was adjusted when required. To avoid EMG sensor disconnection from the human skin during the data acquisition, each sensor was fixed with a strap, as presented in Fig. 1, without introducing erroneous measures.

3D kinematic motion information from the three lower limb joints (hip, knee, and ankle) and pelvis segment was measured at 200 Hz using a motion-capture system with 12 cameras (Oqus, Qualisys – Motion-Capture System, Göteborg, Sweden). Each participant was equipped with the Newington-Helen Hayes marker-set, integrating four more retro-reflective markers placed on the trochanter, medial tuberosity of the femur, medial malleolus, and first metatarsal head to support the calibration process^27^. Overall, 12 pairs of retro-reflective markers were used, as depicted in Fig. 1.

GRF, CoP, and Force Platforms Moments (FPMs) data were provided at 200 Hz, by using six force platforms embedded in the floor: two force platforms FP4060 (Bertec, Ohio, United States of America), two force platforms FP6090 (Bertec, Ohio, United States of America), and two force platform 9281 EA – FP4060 (Kistler, Winterthur, Switzerland). A threshold of 20 N was used to remove noise from the signals.

The placement of the EMG sensors and retro-reflective markers was always performed by the same assessor, who has several years of experience in this field. The accomplishment of palpation procedures to identify anatomical landmarks was performed according to the standards described by Hermens *et al*.^26^ and Tsushima *et al*.^28^, to ensure repeatability in the instrumentation procedure, minimizing the occurrence of occasional and systematic errors.

Moreover, all collected data were time-synchronized through a software trigger. More details about the synchronization procedure can be found in the Technical Validation Section.

### Experimental protocol

All participants were instructed to perform ten rectilinear walking trials on a 10-meter flat surface with six embedded force platforms (as depicted in Fig. 1), at seven different speeds (1.0, 1.5, 2.0, 2.5, 3.0, 3.5, and 4.0 km/h) controlled with a metronome.

In the beginning, the leg, foot length, and body height were measured with a measuring tape, and the body mass was accessed with a balance. Then, the eight EMG sensors were placed on the intended muscles and their functioning was evaluated. Subsequently, two maximum voluntary contractions (MVCs) were performed for each muscle to normalize the EMG data. With the participant seated and performing 90 degrees between all lower limb joints, two MVCs were done for the VL muscle. The participant performed the maximum knee extension while his/her shank segment was manually immobilized (at 90 degrees with the thigh) by an assessor. For BF muscle, the participants laid on a stretcher, in a ventral decubitus position, and with the knee joint at 30 degrees. With the shank manually immobilized by the same assessor, participants carried out maximum knee flexion. Regarding the TA and GAL muscles, participants laid on a stretcher, in a dorsal decubitus. The manual immobilization of the foot was accomplished while the participant was asked to perform maximum *(****i****)* dorsiflexion of the ankle joint (MVC for TA) and *(****ii****)* plantar flexion of the ankle joint (MVC for GAL). For all MVCs, the upper body of the participants was immobilized by a second assessor.

Thereafter, the retro-reflective markers were attached to the participant’s body with double-sided adhesive tape. At this point, the participant was ready to start the data acquisition, as illustrated in Fig. 1.

Firstly, the twelve cameras of the motion-capture system were calibrated according to the manufacturer’s instructions, and the force platforms’ functioning was checked. Then, a standing static calibration trial was performed with the arms crossed in front of the chest, looking forward, and with the feet in a comfortable position, aligned with the shoulders. After the calibration procedure, the participant was ready to perform the walking trials. The participants walked comfortably, looking ahead, and taking steps according to the sound of the metronome. The idea was to avoid the participants seeing the force platforms which could lead the participants to inevitably change their walking patterns according to the force platforms’ position. In case the participants reported they had seen the force platforms, the trial was rejected. For coherency during the data collection, only trials in which the participants step on the first force platform with the right foot were admissible. Additionally, every time that the participant performed two contacts in the same force platform, the trial was also rejected. The first imposed walking speed was 1.0 km/h, using a metronome. Each participant walked during a self-selected time to achieve an adequate familiarization level to the speed. After the familiarization period, each participant performed 10 valid walking trials at the controlled gait speed, as presented in Fig. 1. Once recorded 10 trials, the participant rested for five minutes. The walking speed was incremented, and the participant performed a new habituation trial to become acquainted with the new speed (i.e., 1.5 km/h). Ten valid walking trials were registered, the participant rested for five minutes and a new walking speed was imposed (2.0 km/h). This process was repeated for all the speeds.

### Dataset elaboration

Once the data acquisition has been performed, an identification method of the *Qualisys Track Manager* software (*Automatic Identification of Markers*) was used to preprocess the retro-reflective markers’ coordinates. Despite the retro-reflective markers’ identification method does not allow label duplication, this procedure is not free from defects. Consequently, all marker labeling process was visually inspected by a dedicated assessor. Additionally, to detect marker dropouts, the *X*, *Y*, and *Z* components of each marker trajectory were analyzed. To deal with this phenomenon, the marker trajectory was gap filled through a polynomial interpolation function provided by the *Qualisys Track Manager* software.

The proposed dataset includes ASCII, *.c3d, and *.mat files containing the raw data, namely EMG signals, 3D marker trajectories, GRFs, FPMs, and coordinates of CoP referred to as the global reference frame of the motion-capture system.

The following procedures were addressed to obtain processed data. First, the joint centers were identified as the midpoint between the medial and lateral retro-reflective markers. For example, the knee joint center corresponds to the medial point between the MK and LK markers. Second, segment masses were determined as fixed proportions of body mass based on built-in anthropometric equations in *Visual3D*^29^. Third, we used *Visual3D* software to determine 3D angles and torques for the lower limb joints and pelvis segment, following the C-Motion modeling recommendations^30^. Once computed, the 3D angles were low-pass filtered through a 4^th^ order, zero-lag, Butterworth filter set for a cutoff frequency of 6 Hz^27^. Due to the bidirectional filtering process that minimizes possible phase shifts, data synchronization was not affected. After determining the 3D torques, these data were normalized by the body mass of each participant, being provided the normalized and non-normalized torques. EMG signals were band-pass filtered (4^th^ order, zero-lag, Butterworth filter) with cutoff frequencies of 20 Hz and 450 Hz. Moreover, the envelope of the EMG signals was determined using the Root Mean Square (RMS) value of the signal with a 300 ms movable window, as proposed by Farfán *et al*.^31^.

Furthermore, we used a *Visual3D* software’s function (*Automatic Gait Events*) for automatic detection of the heel-strike events to split all data into individual gait cycles, where the first sample corresponds to the heel-strike instant. The automatic identification performed by the software was analyzed to find incorrect heel-strike detections. For that, the *Z* component of the GRF data was analyzed to determine if the heel-strike detection occurred when the *Z* component started to increase. Every time that an incorrect identification was identified, the heel-strike instant was manually defined. This procedure was useful to gait cycle-normalize to 1001 samples the data of a single gait cycle. During a single trial, the right and left leg performed two and one strides, respectively, since the participants step the first force platform with the right foot. Overall, 1120 trials were collected, containing 1120 and 2240 strides for the left and right leg, respectively. The gait cycle-normalized data, including 3D angles, GRFs, and torques (normalized and non-normalized), filtered, and envelope of the EMG signals for each trial of each participant for the seven walking speeds, are referred to as “processed data” in this dataset, being provided in ASCII and *.mat formats.

## Data Records

All data files are available in *figshare*^32^. The published data are divided into three folders, namely *ASCII files*, *c3d files*, and *MAT files* folders, representing the three formats available. In each folder, the data are organized participant-by-participant according to the *ParticipantID* folder naming convention, where *ParticipantID* represents a folder for each of the sixteen participants that participated in the data collection.

The *ASCII files* folder contains two other folders (*Raw_Data* and *Processed_Data*, with the raw and the processed data saved in the *.txt format). The *MAT files* folder contains two structs (*Raw_Data* and *Processed_Data* with the raw and the processed data saved in the *.mat format and following the same organization as the *ASCII files* folder). The *c3d files* folder contains a folder (*Raw_Data*, with the raw data saved in the *.c3d format and following the same organization as the *ASCII files* folder). Additionally, these three types of folders also contain a text file (*Metadata.txt*) containing the metadata of each participant.

It is noteworthy that all data referred to as “raw data” provide information along the 10 m-flat surface. In contrast, the data presented as “processed data” only correspond to the strides carried out in the force platforms, presenting one and two strides for the left and right legs per trial, respectively.

### Raw data

Raw data enables future processing procedures, such as filtering or resampling. As this study aims to present the collected data per walking speed for each participant, the *Raw_Data* folder of the *ASCII files* and *c3d files* folders is organized onto seven other folders with the following naming convention: *VX*, where *X* can take the values 1, 15, 2, 25, 3, 35, and 4, representing the speeds of 1.0, 1.5, 2.0, 2.5, 3.0, 3.5, and 4.0 km/h, respectively. For each speed, the following six folders are provided: *CoP*, *EMG*, *GRF*, *FPM*, *Trajectories*, and *Properties*, which include the CoP in the force platforms (in m), the EMG signals of the eight muscles (in V), the GRF (in N) and the Moments (in N.m) provided by the force platforms, the trajectories of the 24 retro-reflective markers (in mm), and the units of each data type, respectively. Each one of the first five folders contains the participant’s trials without resampling steps in both ASCII and *.c3d formats. The same organization procedure was followed for the *Raw_Data* struct of the *MAT files* folder, presenting the data in the *.mat format.

For both ASCII and *.mat format files, each trial of the CoPs and FPM data present nineteen columns, where the first column represents the trial duration and the remaining eighteen columns correspond to the 3D data from the six force platforms. The first column is labeled as *Time* and the next eighteen columns are labeled as *FPn_P*, where *(****i****) n* can take the numbers from 1 to 6, corresponding to each one of the six force platforms; and *(****ii****) P* represents the 3D plane and it can take the label *X*, *Y*, or *Z*, representing to the sagittal, coronal, and transversal plane, respectively.

For both ASCII and *.mat format files, each EMG data’ trial presents nine columns. The first column represents the trial duration and the remaining columns correspond to the eight monitored muscles of both legs. Thus, the first column is labeled as *Time* and the subsequent columns are labeled as *L_Muscle*, where (***i***) *L* corresponds to the leg and it can take the nomenclature of *R* (right leg) or *L* (left leg); and (***ii***) *Muscle* represents the BF, GAL, TA or VL muscles.

For both ASCII and *.mat format files, GRF data’ trials contain seven columns, where the first one represents the trial duration, being labeled as *Time*, and the columns are labeled as *L_GRF_P*, where (***i***) *L* corresponds to the leg; and (***ii***) *P* is regarding the 3D plane. Since we defined a threshold of 20 N, an empty cell in the CoP, FPM, and GRF raw data is created for each timestep, while the threshold is not reached. However, the software tool (*Matlab®*) that was used to organize and save all data, automatically set the empty cells as *NaN* values.

**Fig. 2:**
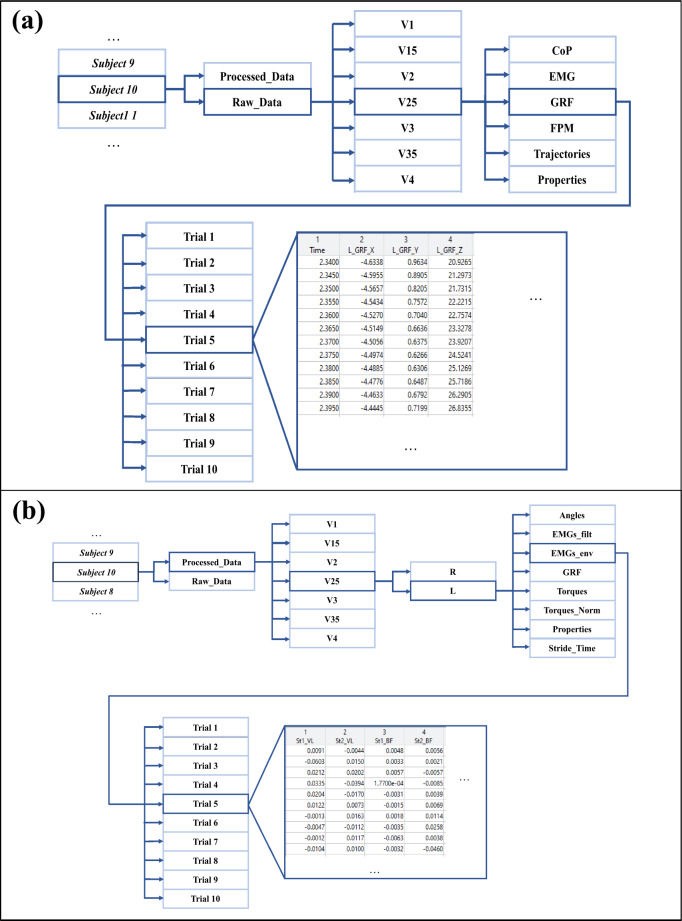
Data organization in the proposed database. Example of (**a**) raw data and (**b**) processed data organization.

At last, for both ASCII and *.mat format files, each trial of the Trajectories data presents the 3D coordinates of each one of the retro-reflective markers presented in Fig. 1(b), labeled as *LMarkerP*, where *L* corresponds to the leg, *Marker* represents each retro-reflective marker and *P* concerns to the 3D plane. Figure 2(a) illustrates the raw data organization for both ASCII and *.mat formats.

### Processed data

The *Processed_Data* folder of the *ASCII files* folder, includes the gait cycle-normalized angles and torques (normalized and non-normalized by the participant’s body mass), GRFs, EMG filtered, and EMG envelope data. Its organization method is similar to the *Raw_Data* folder. The folder of each participant is divided into seven other folders, representing the seven speeds. Each walking speed is split into two new folders, representing the right and left leg. For each leg, the angles (in degrees), torques (N.m), torques normalized by body mass (in N.m/kg), GRF (in N), EMG filtered (in V), and EMG envelope (in % MVC) data are provided per trial in ASCII format files. The units of each data type are presented in the *Properties* folder. Each trial has the information for the gait-cycle normalization, resampled to 1001 samples. For the identification of strides of the right leg (there are two strides per trial), we used *St1* and *St2* to represent the data from the first and second strides, respectively. The information of the stride time is presented in the *StrideTime* folder, in seconds. The same organization procedure was followed for the *Processed_Data* struct of the *MAT files* folder, presenting the data in the *.mat format.

For both ASCII and *.mat format files, each trial’s data of the lower limb joint and pelvis angles and normalized and non-normalized torques is organized in a table. The lines represent the samples and each column is labeled considering the following convention: *L_Local_P*, where (***i***) *L* corresponds to the leg; (***ii***) *Local* represents each lower limb joint along with the pelvis segment and it can take the name of *Ankle*, *Knee*, *Hip* or *Pelvis*. The pelvis angles and torques were determined concerning the virtual lab and considering the distal propagation to proximal of the ground reaction forces; and (***iii***) *P* is regarding the 3D plane.

For each trial of the GRF data stored in ASCII and *.mat format files, the columns are labeled as *L_GRF_P*, where (***i***) *L* corresponds to the leg; and (***ii***) *P* is regarding the 3D plane.

For both filtered EMG and envelope stored in ASCII and *.mat format files, each trial presents eight columns corresponding to the eight monitored muscles of both legs. Each column is labeled as *L_Muscle*, where (***i***) *L* corresponds to the leg; and (***ii***) *Muscle* represents the BF, GAL, TA, or VL muscles.

For both ASCII and *.mat format files, the stride time is provided in a text file and a table, respectively. For both, the lines represent the number of trials performed. The first column (*Trial*) represents the trial number and the second column (*St1_Time*) represents the stride time in seconds, for the left leg. When considering the right leg, a third column is presented, being labeled as *St2_Time*. Figure 2(b) represents the Processed Data organization for both ASCII and *.mat formats.

### Metadata

The *Metadata.txt* file contains the following information: *(****i****) ID*, the participant’s identification defined as *ParticipantX*, where *X* varies from 1 to 16; *(****ii****) Age*, the participant’s age in years; *(****iii****) Gender*, the participant’s gender (*Male* or *Female*, representing male or female, respectively); *(****iv****) Body Height*, the participant’s body height in meters; *(****v****) Body Mass*, the participant’s body mass in kilograms; *(****vi****) Leg Length*, the participant’s leg length in meters; and *(****vii****) Foot Length*, the participant’s foot length in meters.

### Data limitations

During the dataset organization, we observed some data irregularities in particular gait trials, which might affect the dataset interpretation and use. These trials were removed. Table 2 indicates the number of kept trials considering the participant *ID* and walking speed for both raw and processed. This information may be considered before the use of the provided data.

**Table 2:** Number of kept trials for each participant, considering the walking speed variation.

Speed	1.0	1.5	2.0	2.5	3.0	3.5	4.0
ID							
1	10 (7)	10 (8)	10 (9)	10 (10)	10 (9)	10 (8)	7 (7)
2	10 (3)	10 (6)	10 (7)	10 (7)	10 (9)	10 (7)	10 (4)
3	10 (10)	10 (10)	10 (10)	10 (10)	10 (10)	10 (10)	10 (10)
4	9 (9)	10 (10)	10 (10)	10 (10)	8 (8)	0 (0)	0 (0)
5	10 (10)	10 (10)	10 (10)	10 (10)	10 (10)	10 (9)	9 (9)
6	10 (0)	10 (1)	9 (1)	10 (0)	10 (10)	10 (0)	10 (0)
7	10 (0)	10 (0)	9 (0)	10 (0)	10 (0)	10 (0)	10 (0)
8	10 (10)	10 (8)	10 (10)	10 (10)	10 (10)	10 (10)	10 (10)
9	10 (10)	10 (10)	10 (10)	10 (10)	10 (10)	10 (10)	10 (10)
10	10 (9)	10 (10)	10 (10)	10 (10)	10 (10)	10 (10)	10 (10)
11	8 (7)	10 (9)	10 (10)	9 (9)	10 (10)	10 (10)	10 (10)
12	10 (10)	10 (10)	10 (10)	10 (10)	10 (10)	10 (10)	10 (10)
13	9 (8)	10 (10)	10 (10)	10 (10)	10 (10)	10 (10)	10 (10)
14	10 (5)	10 (9)	10 (9)	10 (10)	10 (10)	10 (10)	9 (9)
15	6 (6)	10 (10)	10 (10)	10 (10)	10 (10)	10 (10)	9 (9)
16	10 (10)	9 (9)	8 (8)	4 (4)	3 (3)	5 (5)	10 (10)

The digits within parentheses only concern the number of kept trials for the EMG data while the remaining digits correspond to the number of kept trials for the remaining data (CoP, FPM, GRF, Trajectories, angles, and torques).

Additionally, we identified some general limitations in the dataset. First, the number of participants involved in this study is insufficient to consider the proposed dataset as a reference data for the world young population. Second, the small age range presented by the participants restricts the use of the proposed dataset for a widespread population, covering other age groups. Further, our dataset does not provide EMG data of any hip musculature. Third, this dataset does not include gait transitions, neither other gait activities, such as climbing stairs or ramps since we focused on stable level-ground and continued walking. Thus, the use of the provided gait trajectories as reference trajectories in robotic assistive devices only addresses repetitive level-ground walking therapy at controlled speeds. At last, the data acquisition was conditioned by two external factors: *(****i****)* the use of a metronome to impose a cadence, which may change the gait pattern, according to Bertram *et al*.^33^; *(****ii****)* the requirement of a single step in each force platform.

## Technical Validation

### Data synchronization

For data synchronization, each data acquisition system was connected via cable connectors to a central computer, running the *Qualisys* software. When a new recording started, the *Qualisys* software sent a digital pulse to the EMG and force platform systems, beginning the acquisition in all systems at the same time. The trial recording stopped when another digital pulse was sent to the devices, ending the session at the same instant.

### Kinematic, kinetic, and EMG data

Before starting a trial, we addressed the following procedures. First, all cameras were adjusted and calibrated following the manufacturer’s instructions. Second, the force platforms’ functioning was checked, and a reset was performed to remove the offset introduced by the acquisition software.

**Fig. 3:**
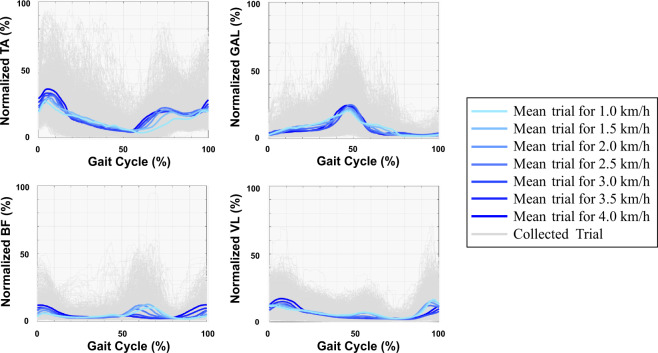
Mean of the muscular activations of TA, GAL, BF, and VL muscles for all participants and trials during a single gait cycle considering the walking speed variation.

**Fig. 4:**
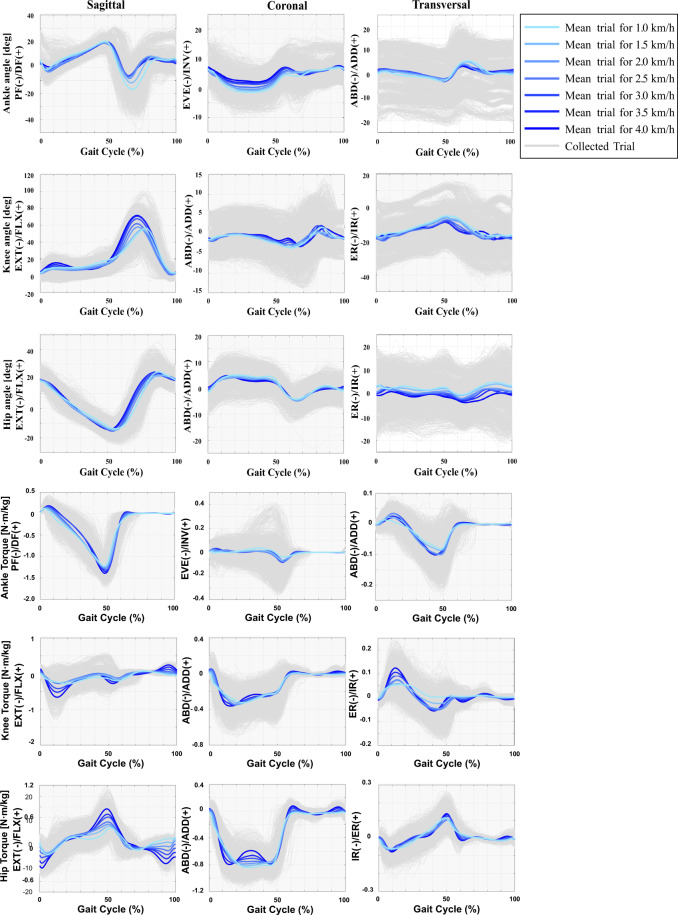
Mean of the ankle, knee, and hip joint angles and torques in the sagittal, coronal, and transversal planes for all participants and trials during a single gait cycle considering the walking speed variation, where: PF – Plantar Flexion; DF – Dorsiflexion; ABD – Abduction; ADD – Adduction; IR – Internal Rotation; ER – External Rotation; EXT – Extension; FLX – Flexion; EVE – Eversion; INV - Inversion.

The retro-reflective markers and the EMG sensors were placed on each participant, as described by Hermens *et al*.^26^ and by Rabuffetti *et al*.^27^. Before each acquisition session, the quality of the EMG signals was verified, and the sensor’s locations were adjusted when required. If the signal was noisy, the skin was cleaned once again with alcohol. Figure 3 illustrates the averaged muscular activation of the TA, GAL, BF, and VL muscles per speed along with the muscular activations of all trials. Additionally, Fig. 4 depicts the mean of all trials per speed of the sixteen participants for the ankle, knee, and hip joint angles and torques in the three planes. In our study, positive values of angles represent (***i***) hip and knee flexion, and ankle dorsiflexion in the sagittal plane; (***ii***) hip and knee adduction, and ankle inversion in the coronal plane; and, (***iii***) hip and knee internal rotation, and ankle adduction in the transversal plane; while negative values represent (***i***) hip and knee extension, and ankle plantarflexion in the sagittal plane; (***ii***) hip and knee abduction, and ankle eversion in the coronal plane; and, (***iii***) hip and knee external rotation, and ankle abduction in the transversal plane. On the other hand, positive values of torque indicate (***i***) hip and knee flexion, and ankle dorsiflexion in the sagittal plane; (***ii***) hip and knee adduction, and ankle inversion in the coronal plane; and, (***iii***) hip external rotation, knee internal rotation, and ankle adduction in the transversal plane; while negative values represent (***i***) hip and knee extension, and ankle plantarflexion in the sagittal plane; (***ii***) hip and knee abduction, and ankle eversion in the coronal plane; and, (***iii***) hip internal rotation, knee external rotation, and ankle abduction in the transversal plane.

### Comparison with public walking datasets

In order to assess the validity of the proposed dataset, we performed a comparative analysis with a public walking dataset with gait speed conditions as similar as possible to the ones of our dataset. The proposed dataset was compared to Bovi *et al*.^12^, which presents a multimodal dataset for a healthy adult population with an age range between 22 and 72 years (43.1 ± 15.4 years). It includes the range of the age of the work herein presented (23.8 ± 2.02 years). Moreover, in Bovi *et al*.^12^, the multimodal data was collected for uncontrolled speeds, being categorized as “walking very slowly”, “walking slow”, “walking medium”, “walking fast”, and “natural walking”. The comparative analysis is limited by the differences in the mean value of the age of both populations and the uncertainty of a specific gait speed value. Consequently, we directly compared data for walking speeds of 4.0 km/h with data acquired for a “natural walking” of the public dataset (Bovi *et al*.^12^) since 4.0 km/h commonly represents a human natural gait speed^22^. The determined Pearson’s correlation ranged from 0.675 to 0.986 and from 0.696 to 0.993 for joint angles and torques, respectively. EMG signals from BF and TA muscles (the muscles in common) presented Pearson’s correlation of 0.828 and 0.724, respectively.

Furthermore, we compared the proposed dataset with the one presented by D. Winter^20^, regarding the effects of walking speed variation in walking dynamics. In D. Winter^20^, the lower limb joint torques were collected from an adult population with a mean age of 25.6 ± 6.2 years, a mean body mass of 69.1 ± 8.8 kg, and a mean body height of 1.75 ± 0.078 m. The collected data were analyzed for three self-selected walking speeds (slow, natural, and fast), in the sagittal plane. In our study, we verified that as the walking speed increases, the ankle dorsiflexion torque, the knee flexion torque, and the hip extension torque also increased. These findings were also reported by D. Winter^20^, conferring robustness to the proposed multimodal dataset. Further, our study was also compared to Fukuchi *et al*.^19^, in which 3D lower limb joint kinematics and kinetics were collected from 42 adult volunteers: 24 young adults with a mean age of 27.6 ± 4.4 years, a mean body height of 171.1 ± 10.5 cm, and mean body mass of 68.4 ± 12.2 kg, and 18 older adults with a mean age of 62.7 ± 8.0 years, a mean body height of 1.62 ± 0.095 m, and a mean body mass of 66.9 ± 10.1 kg. Data were collected at three self-selected walking speeds (slow, natural, and fast) during overground walking. It was found a coherency between the proposed dataset and the one presented by Fukuchi *et al*.^19^ concerning the effects caused by the walking speed in the gait dynamics. Nonetheless, in our study, the behavior of the sagittal and transversal ankle joint angles, and the coronal and transversal knee joint angles in response to the walking speed variation was different from the one reported by Lelas *et al*.^13^, Fukuchi *et al*.^19^, and D. Winter^20^. These findings may be justified by the highly reduced walking speeds innovatively approached in our study. Data collection of all covered speeds followed standards of human gait data acquisition, ensuring the reliability of the observed lower limb joint patterns.

## Data Availability

A *Matlab®* script (*CodeAvailability.m* available in *figshare*^32^) is provided to demonstrate how the dataset can be accessed and how to visualize the processed knee joint angles from a single participant (in this case, Participant 1) in the sagittal plane, for the walking speed of 4 km/h. Moreover, the script also presents an example of creating the graph of the vertical component of the raw GRF for a walking speed of 3 km/h. Additionally, it is provided a simple segment of code to access the stride time of all trials for a speed of 2.5 km/h. To run the provided code, the script must be placed on a folder that contains the *MAT files* folder.
